# Bioelectrical impedance analysis for perioperative water management in adult cardiovascular valve disease surgery

**DOI:** 10.1007/s00595-020-02184-3

**Published:** 2020-12-01

**Authors:** Tatsuya Watanabe, Naomasa Ishida, Munenori Takaoka, Kotone Tsujimoto, Kensuke Kondo, Ryutaro Isoda, Takuro Yukawa, Noriyuki Tokunaga, Atsuhisa Ishida, Takuya Fukazawa, Ichiro Morita, Hideo Yoshida, Masahiko Kuinose, Tomoki Yamatsuji

**Affiliations:** Department of General Surgery, Kawasaki Medical School General Medical Center, 2-6-1 Nakasange, Kita-Ku, Okayama-Shi, Okayama, Japan

**Keywords:** Valve surgery, Bioelectrical impedance analysis, Perioperative water management, Edema index

## Abstract

**Purpose:**

Bioelectrical impedance analysis (BIA) has been used recently to measure the body water of patients with acute heart failure. We used BIA in this study to better understand, and possibly identify a predictive marker for, perioperative water behavior in cardiac surgery patients.

**Methods:**

We measured body water and studied its behavior in 44 patients undergoing surgery for cardiac valvular disease at our hospital. Measurements included the levels of extracellular water (ECW), intracellular water (ICW), and total body water, the edema index (EI), and the ratio of ECW to total body water. The first measured EI was defined as the “preoperative EI” and the maximum as the “peak EI”.

**Results:**

A negative correlation was found between the preoperative EI and the preoperative estimated glomerular filtration rate (eGFR) (*R* = 0.644, *p* < 0.001). Positive correlations were found between the peak EI and the ICU stay (*R* = 0.625, *p* < 0.001), the peak EI and the ventilation time (*R* = 0.366, *p* < 0.01), and the preoperative EI and the ICU stay (*R* = 0.464, *p* = 0.026).

**Conclusion:**

The EI is possibly a predictive marker for perioperative water management in cardiac surgery.

## Introduction

Perioperative water management has a potent influence on both the postoperative course and the development and severity of complications [1–4]. Recently, we evaluated arterial pressure, pulmonary artery pressure, central venous pressure, cardiac index, in–out water balance, arterial gas, and body weight as potential postoperative markers, but found that none of these parameters provided absolute guidance for postoperative water management.

Bioelectrical impedance analysis (BIA) is a noninvasive way of measuring body composition, including water, muscle, and fat [5, 6]. Because it is noninvasive, BIA-measured body muscle weight is now used widely in criteria for the diagnosis of sarcopenia [7–10]. BIA is also used to measure water in animal models in basic research [11]. According to several reports on BIA in clinical studies, the phase angle (PA), one type of BIA readout, shared a correlation with a nutrition marker and was a potential postoperative risk marker for cardiac surgery [12–15]. Studies on the differences in water behavior with some kinds of diuretics found that one readout of BIA, the edema index (EI), being the ratio of extracellular water to total body water, can predict the amount of fluid reduction needed for patients with acute heart failure and chronic renal disease [16–22]. We hypothesized that perioperative water management could be improved by a better understanding of perioperative water behavior through BIA. We further hypothesized that the EI might be a predictive marker for perioperative risk in cardiac surgery. Therefore, the purpose of this study was to use BIA to clarify perioperative water behavior and to find a predictive marker for cardiac surgery.

## Methods

We measured water behavior in patients who underwent cardiac valvular surgery between March, 2018 and October, 2019 at our hospital. We excluded patients who underwent emergency surgery, those with implanted pacemakers, and those for whom measurements could not be done for any reason. Table 1 summarizes the patients’ baseline clinical characteristics. This retrospective study included 44 patients, of whom 26 (59.1%) were males. The average age was 69.79 ± 11.22 years, 9 patients (20.5%) had diabetes, 18 (40.9%) had a smoking history, 21 (47.7%) had hypertension, and 27 (61.4%) had disease classified as NYHA class II–III.

**Table 1:** Baseline characteristics of the patients

Factor	Number or Mean [SD]
Patients	
Age	69.20 (11.48)
Gender	*N* = 26 (59.1%)
Nutrition markers	
PA (degree)	5.30 (1.90)
BMI (kg/m^2^)	22.01 (4.23)
Alb (g/dL)	3.87 (0.64)
Operative characteristics	
Mitral valve replacement	*N* = 2 (4.6%)
Aortic valve replacement	*N* = 13 (29.6%)
Mitral valve repair	*N* = 15 (34.1%)
LA thrombus excision	*N* = 1 (2.3%)
Combined surgery	*N* = 13 (29.6%)
CPB time (min)	170.79 (44.0)
Duration of surgery (min)	326.86 (67.22)
Comorbidities	
Diabetes	*N* = 9 (20.5%)
Hypertension	*N* = 21 (47.7%)
Peripheral arterial disease	*N* = 1 (2.2%)
COPD	*N* = 14 (31.8%)
eGFR (ml/min/1.73 m^2^)	58.9 (25.63)
LVEF (%)	60.11 (9.33)
NYHA class II–III	*N* = 27 (61.3%)

*PA* phase angle, *BMI* body mass index, *Alb* Albumin, *CPB* cardiopulmonary bypass, *LVEF* left ventricle ejection fraction, *NYHA* New York Heart Association functional classification

Values are expressed as absolute numbers and rates for categorical factors, and as the mean and SD in brackets for continuous factors

BIA is a technology that sends a weak electric current through the body to calculate electrical impedance, which in turn measures body components. We obtained eight-polar BIA measurements using an InBody S10 body water analyzer (InBody Japan, Tokyo, Japan) with multiple frequency settings (1, 5, 50, 250, 500 and 1000 kHz). Measurements at multiple frequencies allow for an accurate estimation of both intracellular and extracellular water content inside and outside cells separately [19]. Only impedance is used to calculate body composition results, meaning that no empirical estimations such as age or gender are used or required by the S10 to calculate body composition.

The InBody S10 can analyze the body composition and body water of patients who are immobile or have amputated limbs, via attachable electrodes, even while the patient is prone. This could be beneficial for bedridden patients in the ICU to monitor changes in body and fluid composition precisely (InBody US homepage, http://inbodyusa.com). The InBody S10 was used to measure both pre- and postoperative water behavior, in addition to other traditional monitoring. The preoperative data were measured soon after anesthetic induction, and then once every 6 h until postoperative day 2. From day 3, measurements were taken either twice a day in the intensive care unit or once a day in the general ward.

Perioperative clinical data were collected pre- and postoperatively for atrial fibrillation, pleural effusion, perioperative water in–out balance, ventilation time, and the length of ICU stay. Other more conventional water management data collected included body weight, P/F ratio, 2-hourly urine output, and 2-hourly intake and output fluid balance. The InBody S10 was used to measure the levels of extracellular water (ECW), intracellular water (ICW), total body water (TBW), the edema index (EI, the ratio of ECW to TBW) and phase angle (PA, the arctangent value of the directly measured ratio of Reactance (Xc) to Resistance (R)). The phase angle does not depend on conventional regression equations of body composition and has been reported as an objective prognostic marker of disease severity and frailty [13, 14]. The first measured EI was defined as the “preoperative EI” and the maximum EI as the “peak EI”. Figures 4, 5 show the mean BIA data as successive data. Each patient’s BIA data and conventional water management data were analyzed for correlations between the preoperative and peak data, using linear regression analysis, to identify correlations among parameters.

This study was conducted with the approval of the ethics committee of Kawasaki Medical School, and all patients provided informed consent. The study is registered with the clinical trials registry of the Kawasaki Medical School Information Network, Number UMIN000029876.

### Statistical analysis

Linear regression analyses between the preoperative or peak BIA data, which included ECW, ICW, TBW, and EI, and all parameters, namely age, BMI, change of body weight, eGFR, CPB time, duration of surgery, hemoglobin, creatinine, BNP, albumin, ventilation time, and ICU stay, were conducted to identify correlations. We tried to find correlations between BIA data and any parameter, using simple linear regression analysis, and also between outcomes and BIA data. A two-tailed p value less than 0.05 was considered significant. Statistical analysis was performed with R 3.6.0 (the R Foundation for Statistical Computing, Vienna, Austria).

## Results

Preoperative blood tests showed the following results: hemoglobin, 13.15 ± 2.27 g/dL; hematocrit, 39.20 ± 8.38%; creatinine, 1.31 ± 1.49 mg/dL (median: 0.87); BNP, 395 ± 844 ng/dL (median: 216); eGFR, 57.51 ± 25.71 ml/min/1.73 m^2^ (median: 58.05); and albumin, 3.90 ± 0.62 g/dL. The recorded mean value measurements were as follows: CPB time, 172.20 ± 43.43 min; duration of surgery, 333.15 ± 70.61 min; ventilation time, 4.65 ± 7.18 h (Median: 2 h); and ICU stay, 3.08 ± 1.23 days (Median: 3 days).

**Fig. 1:**
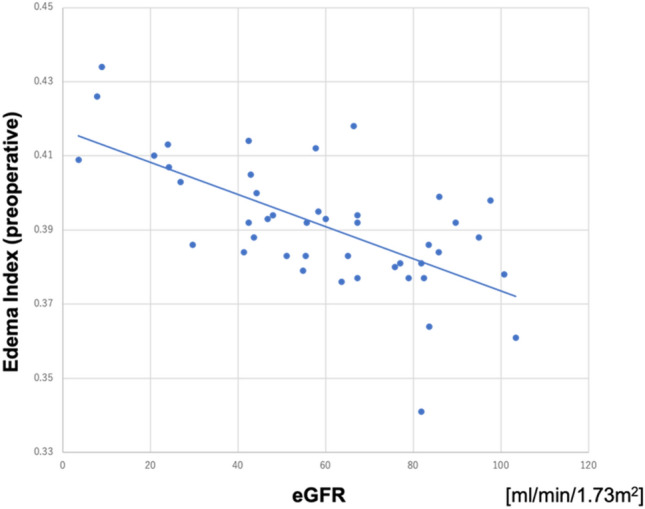
Relationship between the edema index (EI) and the estimated glomerular filtration rate (eGFR). There was a negative correlation between the preoperative EI and preoperative eGFR (*R* = 0.644, *p* < 0.001)

**Fig. 2:**
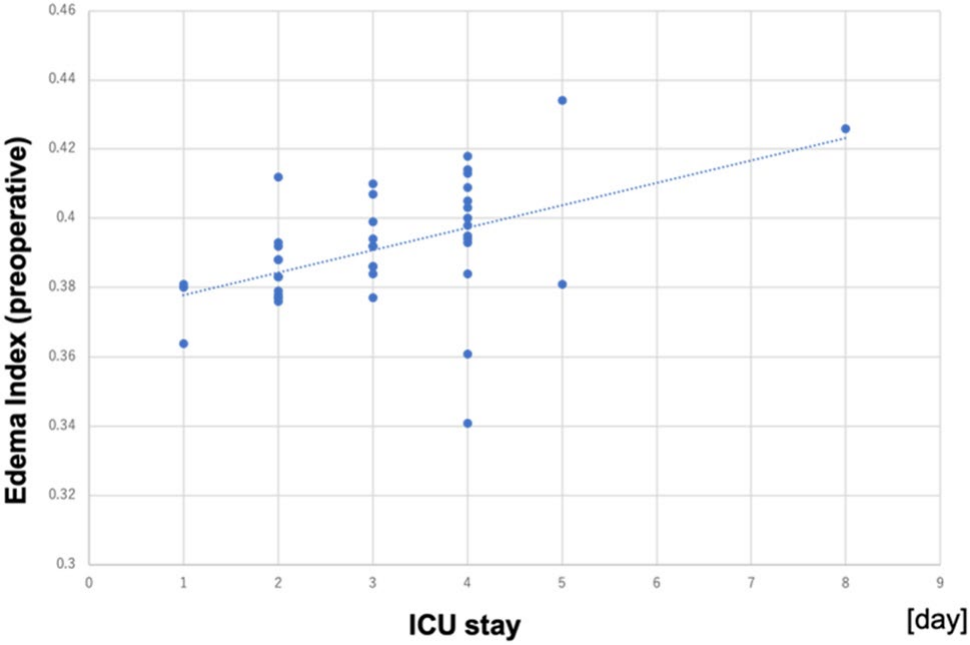
Relationship between the edema index (EI) and the duration of the intensive care unit (ICU) stay. There was a positive correlation between the preoperative EI and the duration of the ICU stay (*R* = 0.625, *p* < 0.001)

**Fig. 3:**
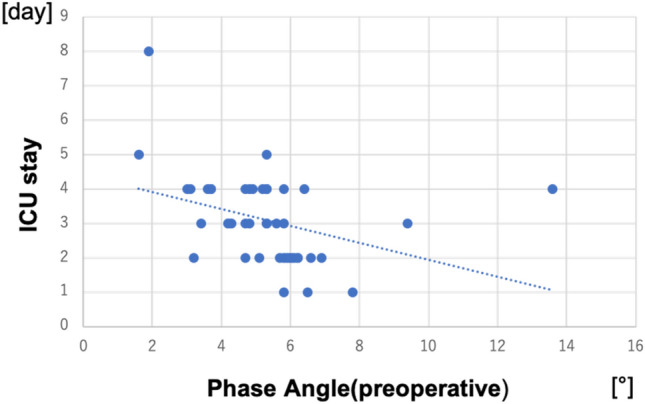
Relationship between the preoperative phase angle and the duration of the ICU stay. There was a weak negative correlation between the preoperative phase angle and the duration of the ICU stay (*R* = 0.118, *p* = 0.014)

A negative correlation was found between the preoperative EI and the preoperative eGFR (*R* = 0.644, *p* < 0.001; Fig. 1). The preoperative EI was correlated with hemoglobin (*R* = 0.541, *p* < 0.001), creatinine (*R* = 0.433, *p* < 0.01), albumin (*R* = 0.512, *p* < 0.001), and BMI (*R* = 0.340, *p* = 0.014). Positive correlations were found between peak EI and ICU stay (*R* = 0.625, *p* < 0.001) and between peak EI and the ventilation time (R = 0.366, *p* < 0.01). The length of ICU stay was also correlated positively with preoperative EI (*R* = 0.464, *p* = 0.026) (Fig. 2), peak TBW (*R* = 0.348, *p* = 0.011), peak ECW (*R* = 0.261, *p* = 0.048) and peak ICW (*R* = 0.378, *p* = 0.0065). The phase angle had a weakly negative correlation with the ICU stay (*R* = 0.118, *p* = 0.014) (Fig. 3). Body weight changes showed only a weak correlation with EI (*R* = 0.281, *p* = 0.036), but no correlation with ECW(*R* = 0.132, *p* = 0.616), ICW(*R* = 0.017, *p* = 0.320), or TBW(*R* = 0.009, *p* = 0.413). Body weight changes also did not show any correlations with the ICU stay (*R* = 0.093, *p* = 0.248) or ventilation time (*R* = 0.139, *p* = 0.670).

We also measured the TBW (preoperative) as 30.35 ± 8.68 kg, the ICW (preoperative) as 18.52 ± 5.54 kg, the ECW (preoperative) as 11.82 ± 3.19 kg, the EI (preoperative) as 0.391 ± 0.016, the TBW (peak) as 34.33 ± 8.37 kg, the ICW (peak) as 20.77 ± 5.20 kg, the ECW (peak) as 13.77 ± 3.13 kg, the EI (peak) as 0.409 ± 0.013, and the phase angle (PA) as 5.30 ± 1.90°. The operations performed included 13 aortic valve replacements, 2 mitral valve replacements, 15 mitral repairs, 13 combined cases, and 1 left atrium thrombectomy.

**Fig. 4:**
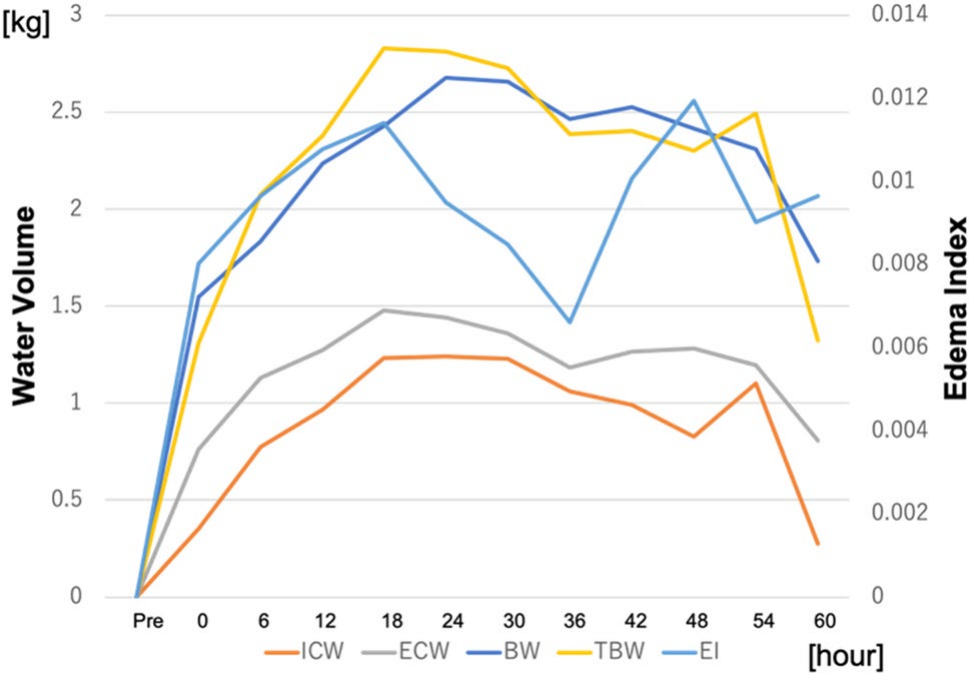
Successive data of the bioelectrical impedance analysis (BIA) and body weight. This figure shows the mean values of the changes in successive BIA data for all patients. The extracellular water (ECW) and intracellular water (ICW) increased soon after the operation before finally decreasing. The ECW increased more than the ICW and decreased sooner than the ICW, whereas the edema index (EI) increased soon after the operation, decreased once, increased again, and then finally decreased

**Fig. 5:**
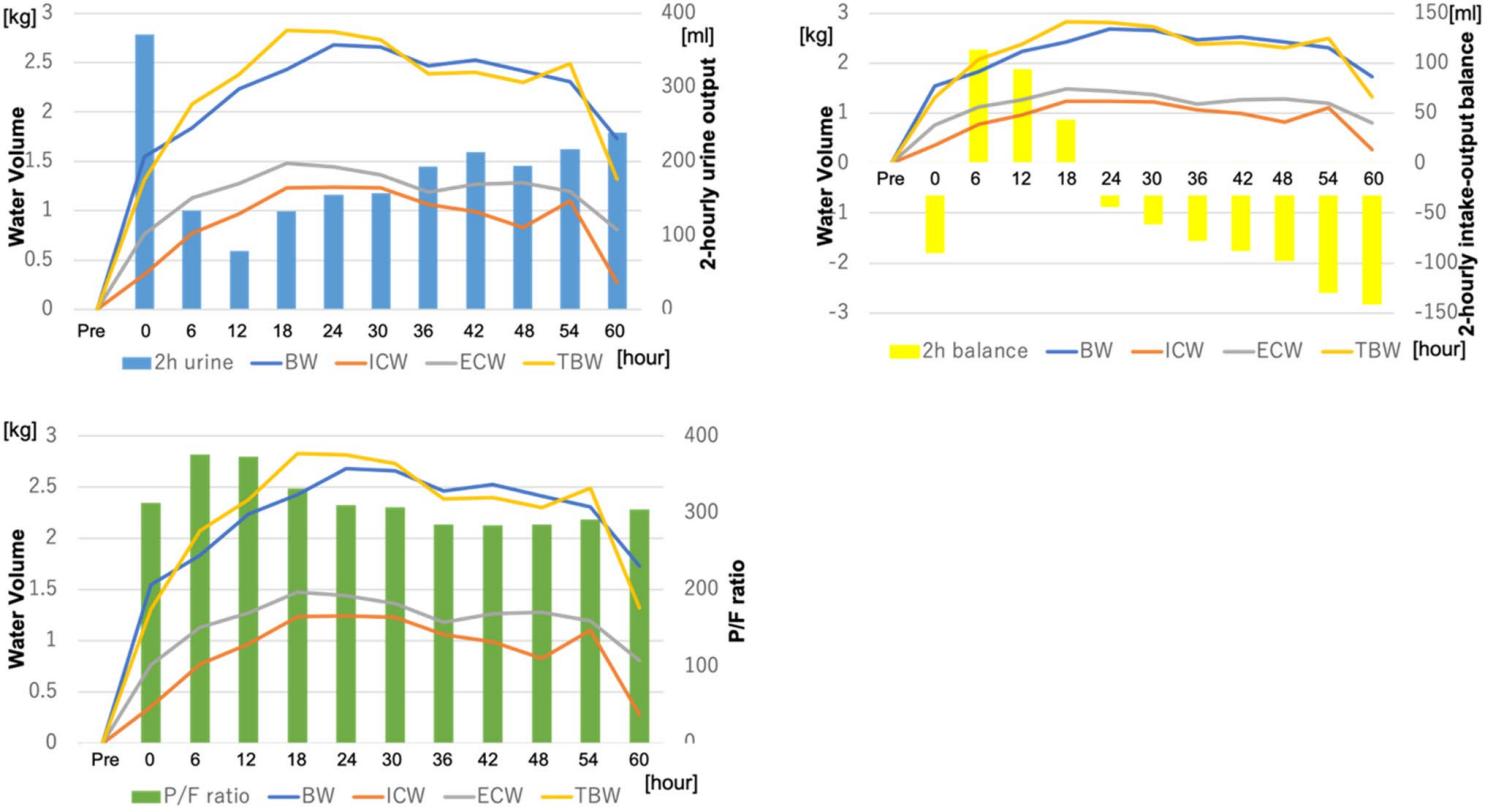
Successive data of the bioelectrical impedance analysis (BIA), 2-hourly urine, 2–hourly intake–output balance and the P/F ratio. This figure shows the mean values of successive BIA data compared with 2-hourly urine output, 2-hourly intake–output balance and the P/F ratio. Urine output was highest 2 h after the operation, then decreased greatly, and increased gradually as the water volume increased. The 2-hourly fluid balance was negative soon after the operation, became positive over the next 6 h, and finally became negative again as the urine output increased. The P/F ratio decreased as the water volume increased and recovered as the water volume decreased

**Fig. 6:**
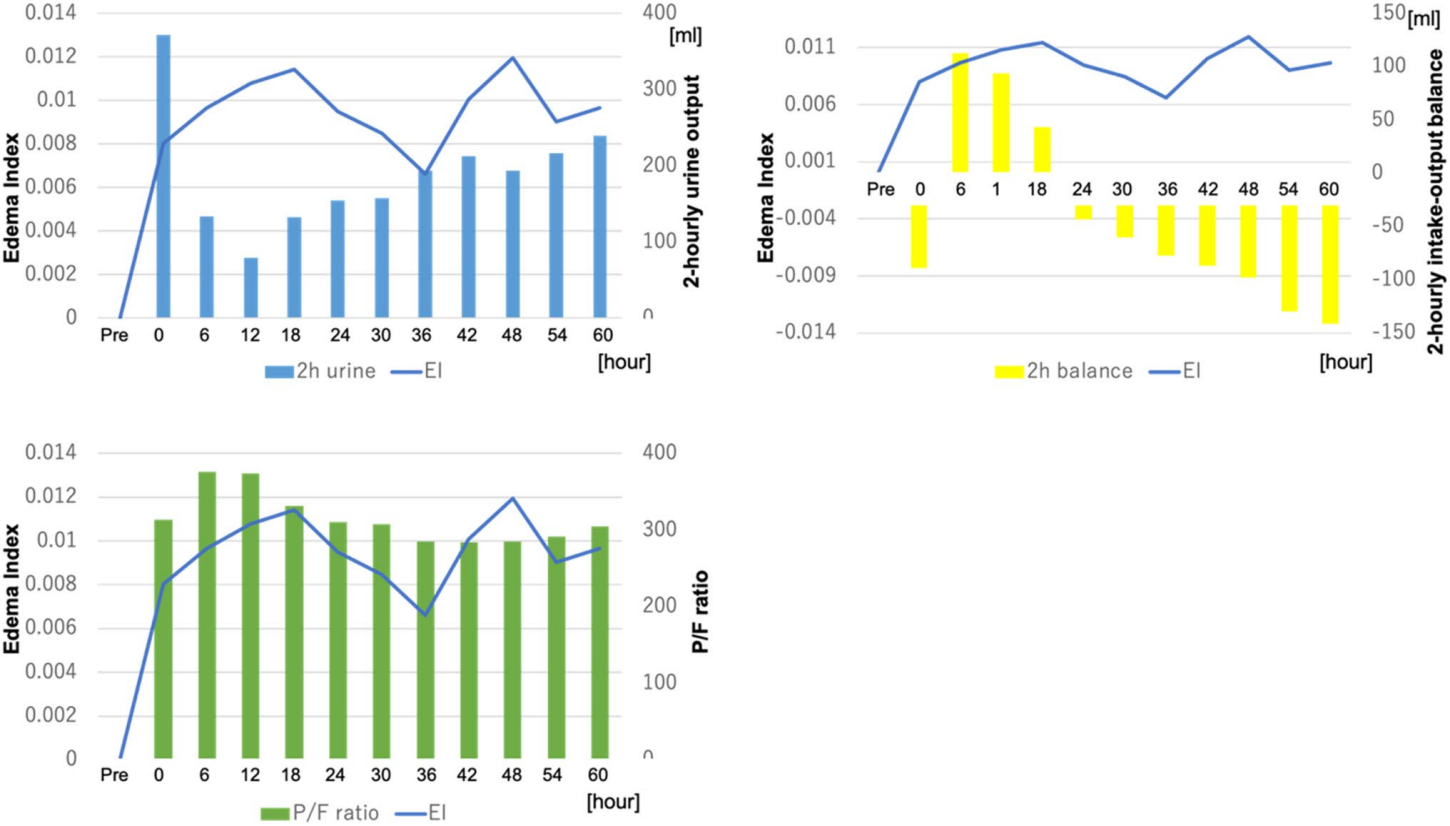
Successive data of the edema index (EI), 2-hourly urine, 2-hourly intake–output balance and the P/F ratio. This figure shows the mean value of the successive EI and 2-hourly urine output, 2-hourly intake–output balance and the P/F ratio and the relationships between the EI and other parameters

Figures 4, 5, 6 show a successive comparison of the mean values of the BIA data and body weight, 2-hourly urine output, fluid intake and output balance, and the PaO_2_/FIO_2_ (P/F) ratio, which are all currently used as postoperative water balance markers. Urine output was highest 2 h after surgery, decreasing greatly and then increasing gradually as water volume increased. The 2-hourly fluid balance was negative soon after surgery but became positive over the next 18 h and then finally became negative again as the urine output increased. The P/F ratio decreased as water volume increased and recovered as water volume decreased.

## Discussion

BIA has been applied for the water management of hemodialysis patients and also recently for the water management of heart failure and cardiac surgery patients [16–22]. The purpose of this research was to assess perioperative water behavior in patients undergoing cardiac surgery. To our knowledge, this research is the first attempt at assessing changes over the perioperative course using BIA. This observational study assessed perioperative water behavior and found that the EI may be a potential future perioperative water management marker for cardiac surgery.

As shown in Fig. 4, the ECW and ICW increased soon after the operation, before finally decreasing. The ECW increased more than the ICW and decreased sooner than the ICW, whereas the EI increased soon after the operation, then decreased once, increased again, and then finally decreased. The mean body weight, which is used as a marker of water balance, behaved similarly to the TBW. This figure does not compare each individual patient’s data, as the means were used to find correlations; however, body weight changes did not show any correlation to the TBW, ECW, or ICW. Body weight also had no correlation with the duration of ICU stay or ventilation time. Therefore, BIA data such as the EI is possibly a better water management predictor than body weight.

Previous studies found that the EI was a useful marker for heart failure [16–18] and the phase angle was a predictive marker for cardiac surgery [12–15]. The EI shows the ratio between intra- and extracellular water. We suspect that a postoperative increase of the EI reflects increasing water in the extracellular space, including the interstitial third space, caused by the invasiveness of surgery. Both intra- and extracellular water increased, as shown in Fig. 4, but the accumulation of fluid in the interstitium is thought to be the cause of the elevated EI. The preoperative EI was correlated with preoperative hemoglobin, albumin, creatinine, and BMI, but the preoperative EI and eGFR values had the strongest correlations. Although the preoperative ICW and eGFR showed weak correlation, and the preoperative ECW and TBW did not show any correlations, the preoperative EI showed a stronger correlation with eGFR. These data suggest that renal function plays a role in maintaining the intra- and extracellular water balance.

When making the decision about moving a patient to a general surgical ward, the ICU doctors discuss the patient’s circulatory dynamics, respiratory condition, and renal function stability. This study used similar criteria to exclude patients who remained in the ICU longer than average because of central nervous system complications, since they focused solely on water behavior. A high preoperative EI and a peak EI meant more extracellular water, which we suspect from the perioperative clinical course reflects extra water in the third interstitial space. Although the peak TBW, peak ICW, and peak ECW were all correlated with the duration of ICU stay, the peak EI showed an especially strong correlation with the ICU stay. We suspect that the balance between extra- and intracellular water is the most important factor for postoperative water management. These results might be reflected in the fact that our postoperative management focused mainly on water behavior. While the changes between the preoperative EI and the peak EI did not show a correlation with the ICU stay or ventilation time, we speculate that patients with a lower preoperative EI have more extra space to accumulate water, whereas those with a higher preoperative EI have less space to accumulate water, which could also explain why the changes between the pre- and postoperative EI did not reflect the postoperative course.

Ringaitiene et al. [13, 15] reported that the phase angle reflects the preoperative nutritional state of cardiac surgery patients and that patients with a lower PA needed more blood transfusions and had more complications. In our study, PA had a weak negative correlation with the ICU stay, so patients with a lower PA tended to stay in the ICU longer. These results were similar to those of Ringaitiene et al. Patients with a higher preoperative EI, a lower PA, and a higher peak EI are more likely to have a longer ICU stay. Considering those results, water management for patients with a higher preoperative EI with the aim of lowering peak EI may shorten the time spent in the ICU. We will research the effect of using water management to lower peak EI prospectively.

### Limitations

The major limitation of this study is that it was an observational study with a limited number of patients in a single institute. In this study design, the BIA was measured several times a day in the ICU, but only once a day in the general ward. This caused some measurement timing fluctuations, especially after postoperative day 3. Some measurements were missed because of the condition of the patient at the time. In this study a few patients with a history of hemodialysis were included, and as their water management was closely related to their dialysis, the data for these patients were not included in the graphs.

## Conclusions

The EI may be an effective marker for the perioperative water management of patients undergoing cardiac surgery. The phase angle could also be a risk predictive factor, as reported previously.

## Data Availability

Not applicable.

## Abbreviations

BIA: Bioelectrical impedance analysis
ECW: Extracellular water
ICW: Intracellular water
TBW: Total body water
EI: Edema index
PA: Phase angle
ICU: Intensive care unit
NYHA: New York Heart Association
eGFR: Estimated glomerular filtration rate
